# A Short Review

**Published:** 1865-12

**Authors:** 


### A SHORT REVIEW.

As the dental profession justly claims rank with the learned professions, it is important that contributors to its journals cultivate a good style of writing.

The author of the first article (of over four pages that might readily have been condensed to less than two) in the Register for the current month, after asserting that he is a "*modest*" man — the italics in the quotations I make are his own — gives us a specimen of diffusive style, narrating that he, "a tooth-carpenter" once "rode forty miles" to have "an old sentinel" removed. He "honestly" says he had "*great happification*" in being shown the "villianous offender" without enduring the pain he "apprehended, and had experienced, from the *hacking* and *gouging* of the gum-lancet."

Of course a "first class operator" will procure forceps "well adapted to the teeth."

Disclaiming the charge of being a "*hobby rider*" the author tells us he has "rode it"— "the practice" of "the new system" of extracting teeth without using the lancet — "into the mouths of many, very many," by the aid of said "*hobby.*" The repeated use of the French language indicates that he is a gentleman familiar with polite literature; but he is unfortunate in the use of his native tongue, as well as in the coinage of such words as "*happification.*" Dentists sometimes employ the word troubled to express what he means by "bothered." They cut or divide the gums around teeth, instead of "*hacking* and *gouging* around the gums;" and as to "pulling teeth," they remove or extract them; nor do they enhance their reputation for veracity by reporting that their patients say, when only forceps are used, their teeth are extracted with "no pain at all."

An egotistical or redundant style of writing is not often improved by omitting the nominative pronoun I, abbreviating are not, to "a'nt", nor by a repetition in the same sentence, as in the following: "Am satisfied, therefore, if the practice is adopted by those of the profession, *who can possess themselves* — can keep cool — that a'nt bothered with the shakes, I repeat, *can possess themselves*, so that they can watch closely what they are doing, when pulling a tooth, they will rarely have use for the gum-lancet again."

After the hemorrhage has ceased he touches the gums with "arnica, and camphor," (tinctures of course, although he does not say so,) and recommends their use to the patient, finding them "most excellent in curing up the gums." Would they not be quite as good for curing them down?

Nov. 24th, 1865. OBSERVER.

